# Differences in emotion regulation components underlie the sexual orientation disparity in depressive symptoms: a prospective, Population-based study of young adults

**DOI:** 10.1007/s00127-025-02974-5

**Published:** 2025-09-05

**Authors:** Ilana Seager van Dyk, Caroline G. Rutherford, John E. Pachankis, Richard Bränström, Mark L. Hatzenbuehler

**Affiliations:** 1 Department of Social & Behavioral Sciences, Yale School of Public Health, New Haven, CT USA; 2 School of Psychology, Massey University, P.O. Box 756, Wellington, 6140 New Zealand; 3 Department of Epidemiology, Columbia University Mailman School of Public Health, NY, USA; 4 Department of Clinical Neuroscience, Karolinska Institutet, Stockholm, Sweden; 5 Department of Psychology, Harvard University, Cambridge, MA USA

**Keywords:** Emotion regulation, Sexual minority, Mental health disparities, Development, Depression

## Abstract

**Purpose:**

Hatzenbuehler’s psychological mediation framework proposes that difficulties in emotion regulation (ER), which are driven in part by excess exposure to stigma-related experiences, contribute to sexual orientation-related mental health disparities. However, existing research on the framework has largely focused on a small number of ER variables in non-probability samples.

**Methods:**

To address these limitations, we examined whether a large complement of ER components mediates the prospective association between sexual minority status and depressive symptoms, using longitudinal data from a population-based sample of 1,208 Swedish young adults (aged 18–35). Data were collected in 2020 (ER, depressive symptoms) and 2021 (depressive symptoms). Participants completed 12 measures of ER, spanning a diverse array of ER constructs (e.g., emotional awareness, cognitive reappraisal, access to ER strategies).

**Results:**

Sexual minorities exhibited significantly more ER difficulties on nine out of the 12 ER components, and higher depressive symptoms, compared to heterosexuals. Eight of the 12 ER components independently mediated the association between sexual minority status and increases in depressive symptoms one year later, and two components (brooding rumination, difficulty identifying positive emotions) mediated this relationship when all 12 ER components were entered into the model simultaneously.

**Conclusion:**

These findings provide evidence from a population-based, longitudinal study that a wide range of ER factors underlie sexual orientation-related disparities in depressive symptoms during a developmental period of heightened risk.

## Introduction

Sexual minority individuals experience disproportionately high rates of psychopathology, including depression, relative to heterosexual individuals [1, 2]. The psychological mediation framework [3] proposes that emotion regulation (ER)—which encompasses the set of strategies that individuals use to manage which emotions they have, when they have them, and how they express them [4]—is an important mediator underlying sexual minority individuals’ disproportionately high risk of psychopathology. The construct of ER has gained significant attention in recent years as a transdiagnostic mechanism underlying both internalizing and externalizing psychopathology [5].

According to the psychological mediation framework [3], difficulties in ER result in part from sexual minorities’ excess exposure to stressful identity-related experiences (sometimes called minority stressors; e.g., discrimination [6, 7]), as compared to heterosexuals. As a result of these experiences, sexual minorities engage in increased self-monitoring and vigilance for threat, both of which can potentiate ruminative self-focus [3] — a common feature of internalizing conditions like depression and generalized anxiety [8]. This notion that stigmatizing environments impact mental health via emotion processes is reflected in more recent theories suggesting that, for example, emotion invalidation can lead to less clarity about one’s own emotions and increased emotion dysregulation [9], and that the presence of rejecting parents can damage the emotion socialization process that leads to the development of healthy ER among sexual minorities [10].

Motivated by the psychological mediation framework [3], studies have sought to examine associations between stigma-related stressors and ER, between ER and mental health problems, and the mediating role of ER in the association between stigma-related stressors and mental health problems among sexual minority individuals. For instance, heightened ER difficulties among sexual minority individuals are associated with stigma-related stressors [11], including identity-based rejection sensitivity [12], internalized homonegativity [13], childhood trauma [14], intimate partner violence [14], and experiences of homophobia and biphobia [15]. Likewise, studies have found support for the association between ER and mental health outcomes among sexual minority individuals, including internalizing symptoms [12, 13, 16], alcohol and drug use [12, 17, 18], and sexual compulsivity [13, 19]. Studies have also found support for the mediating role of ER in the association between stigma-related stressors, such as discrimination, and various adverse mental health outcomes, including problem drinking, depression, anxiety, non-suicidal self-injury, and binge-eating, among sexual minorities across genders [13, 17, 20–22].

Although the above studies have identified the potential role of ER in sexual minority mental health, this existing research is limited by two important methodological features. First, the vast majority of research on ER in sexual minority populations has operationalized ER as a unitary phenomenon [14, 19], typically relying on a total score of an ER inventory, such as the Difficulties of Emotion Regulation Scale (DERS [23]). However, decades of research reveal that not all ER components are equal. In fact, meta-analytic findings indicate that certain ER components (e.g., rumination, suppression, avoidance, problem-solving) are more strongly related to psychopathology than others (e.g., acceptance, reappraisal), and that associations between specific ER components and psychopathology depend on the type of psychopathology (e.g., rumination has stronger effects on depression and anxiety than on substance use and eating pathology [8]).

Second, studies have thus far not directly compared sexual minority and heterosexual individuals’ endorsement of a large complement of ER components utilizing population-based sampling. A few studies using non-probability sampling find that sexual minority individuals exhibit more general ER difficulties than heterosexuals [21, 24–27]. Other studies have examined possible sexual orientation-related differences in a small number of specific ER components, such as emotional awareness [26, 28], rumination [28], cognitive reappraisal [29], and expressive suppression [29]. However, without examining a wide range of ER components in a population-based sample, the field lacks a comprehensive understanding of the scope and generalizability of sexual orientation-related differences in ER and their role in contributing to sexual orientation-related disparities in mental health. For example, while difficulties identifying and describing emotions (often called alexithymia) are associated with increased rates of anxiety and depression [30, 31], no research designs suitable for the purpose have compared these ER components in sexual minorities and heterosexuals [32]. In addition, non-representative sampling of sexual minority individuals introduces selection biases—including overrepresenting those with a monosexual identity (vs. those with a bisexual or pansexual identity); those who have disclosed their sexual orientation; those who are actively involved in sexual minority communities; those living in urban areas; those with higher incomes, more education, current employment, and greater number of sex partners; and those experiencing more suicidal ideation, alcohol use, and substance use [33–35]. Some of these factors are themselves determinants of ER components [36, 37], which may lead to biased estimates of the relationship between sexual orientation, ER, and mental health. Thus, population-based sampling can enhance both internal and external validity regarding the study of sexual orientation differences in ER and of whether these differences underlie sexual orientation-related mental health disparities.

The present study sought to overcome methodological limitations of existing studies by using longitudinal data from a large, population-based sample of sexual minority individuals and a comparison sample of heterosexual young adults (ages 18–35). Young adulthood is a period of heightened risk for the onset of numerous emotion-related mental health problems, including depressive disorders [38], and thus represents a critical developmental period during which to explore ER differences and their potential mediating role in explaining sexual orientation-related disparities in depressive symptoms.

Our first aim was to investigate sexual orientation differences in a wide range of ER components. We selected 12 ER components that included both those examined in non-probability samples described above, as well as alexithymia-related components, given the relative lack of attention to these ER components despite the importance of understanding one’s own emotions in order to choose a regulatory response [39]. All 12 ER components selected map onto prominent ER theories [4, 39] (e.g., all four stages of the extended process model of ER are represented in this study). Based on the extant literature, we anticipated that sexual minority individuals, relative to heterosexual participants, would have a higher prevalence of all 11 individual ER components typically associated with psychopathology (e.g., rumination, expressive suppression) and a lower prevalence of the ER component associated with adaptation (i.e., cognitive reappraisal), consistent with prior research using non-probability samples [26, 28, 29]. Our second aim was to test the role of ER difficulties in predicting sexual orientation differences in depressive symptoms one year later, controlling for prior symptoms. Based on existing research [26, 28, 29], we predicted that the 11 individual ER components typically associated with psychopathology (e.g., rumination, expressive suppression) would mediate the link between sexual orientation and depressive symptoms, with sexual minorities exhibiting higher ER difficulties that in turn predict higher depressive symptoms one year later.

## Transparency and openness

We report how we determined our sample size, all data exclusions, all manipulations, and all measures in the study. All data were produced under the Swedish Statistics Act and the European Union Data Protection Regulation, according to which privacy concerns restrict the availability of personal data for research. Aggregated data can be made available by the authors, subject to ethical vetting. Enquiries should be made to plus@cns.ki.se. The code for these analyses is publicly available and can be accessed at: https://osf.io/mhsyp/?view_only=378179dda995439e975b18d1db013a20. This study was not pre-registered. This study was approved by the Stockholm Regional Ethical Review Board and the Swedish Ethical Review Authority (2018/1517-31; 2019–06313; 2023-04675-02), as well as the Yale University Human Subjects Committee (No. 2000024703).

## **Method**

### Participants

Participants were drawn from the Pathways to Longitudinally Understanding Stress (PLUS) study, a population-based, longitudinal cohort of young adults in Sweden. The PLUS cohort was recruited among participants from the Swedish National Public Health Survey (SNPHS) conducted by the Public Health Agency of Sweden, for which the sampling frame included all individuals living in Sweden at the time of each survey. To create the PLUS cohort, we invited all 2,943 young adult participants (ages 18–35) who identified as non-heterosexual in the 2015, 2016, and 2018 SNPHS, along with a random sample of 2,943 heterosexual participants in the same age range. For full details of the PLUS study sampling method, see [40].

The current study used data from Wave 2 (conducted in 2020) and Wave 3 (conducted in 2021) of the PLUS cohort, as Wave 1 (conducted in 2019) contained limited ER data. Participants were included if they responded to both Wave 2 and Wave 3. The Wave 2 sample (*N* = 1,679, 75.6% of participants who completed Wave 1), in which the ER and depressive symptoms questions were administered, consisted of 951 heterosexual and 728 sexual minority individuals who completed at least 10% of survey questions, with an average age of 27.12 (*SD* = 5.03; range 18–35). Of the Wave 2 sample, 1,208 participants (41.0% of participants who completed Wave 1) completed measures of depressive symptoms in Wave 3. The final analytic sample for this study (*N* = 1,208) therefore consisted of 667 heterosexual and 541 sexual minority participants, whose demographic characteristics are described in Table 1. Participants were included in the sexual minority group if they selected a minority sexual orientation (e.g., lesbian, gay, something other than “heterosexual”) on a series of multiple-choice questions about their sexual orientation and indicated that they felt comfortable being referred to as “LGBTQ+” [40].

**Table 1:** Demographic characteristics of the analytic sample by sexual orientation group

		Full sample	Heterosexuals	Sexual minorities
Unweighted n		1208	667	541
Age (years)		27.22 (5.05)	27.76 (4.98)	26.57 (5.07)
Sexual orientation	Straight/heterosexual	55.2%	100.0%	–
	Lesbian or gay	10.7%	–	23.8%
	Bisexual	28.4%	–	63.4%
	Queer	1.1%	–	2.4%
	Pansexual	1.2%	–	2.8%
	Asexual	0.8%	–	1.8%
	Demisexual	0.2%	–	0.5%
	Other	0.4%	—	0.9%
	I don’t know	2.0%	—	4.4%
Gender identity	Man	28.0%	33.9%	20.7%
	Woman	69.7%	66.0%	74.3%
	Trans man	0.4%	0.1%	0.7%
	Trans woman	0.2%	–	0.6%
	Genderqueer/gender non-conforming	1.2%	–	2.6%
	Other	0.5%	–	1.1%
Assigned sex at birth*	Male	28.1%	33.4%	21.6%
	Female	71.9%	66.6%	78.4%
Live in Sweden	Yes	99.2%	99.4%	98.9%
	No	0.7%	0.5%	1.1%
	Missing	0.1%	0.1%	–
Employment	Employed	64.9%	71.1%	57.3%
	Disability	3.1%	1.9%	4.4%
	Unemployed/Student	32.1%	27.0%	38.3%
Education	High school diploma or less	45.0%	42.9%	47.7%
	Associate’s or 4-year college degree	14.5%	12.3%	17.2%
	More than a 4-year college degree	40.1%	44.2%	35.1%
	Missing	0.3%	0.6%	–
Income	< 9999 SEK/month	26.4%	20.8%	33.3%
	10,000–19,999 SEK/month	27.2%	25.6%	29.2%
	20,000–29,999 SEK/month	18.6%	18.0%	19.4%
	> 30,000 SEK/month	27.5%	35.2%	18.1%
	Missing	0.2%	0.3%	–
Region	Large central city (pop. 250 K+)	36.4%	37.5%	35.1%
	Medium size city (pop. 50–250 K)	30.5%	30.1%	31.1%
	Small city (pop. 10–50 K)	18.2%	17.5%	19.0%
	Town, village, or unincorporated area	14.7%	14.7%	14.8%
	Missing	0.1%	0.1%	–

Note. * = Measured at baseline; all others were measured at Wave 2. For age, Mean (SD). Age range for the full sample at baseline was 18–35 years. Pop. = population. All participants included in this Table completed both Wave 2 and Wave 3 of data collection. For cultural reasons, it was deemed inappropriate to collect information about race and ethnicity. In Sweden, information about race and ethnicity is not monitored in population surveys but more commonly information about countries of birth and migration history is collected

### Procedure

After providing informed consent, participants completed a battery of questionnaires related to ER processes and depressive symptoms (described in further detail below). Participants received a gift card of 100 SEK for their participation at each assessment wave.

## Measures

### Emotion regulation

#### Perth Alexithymia Questionnaire (PAQ)

Three subscales of the PAQ [41] were administered: the 4-item *Difficulty Identifying Negative Feelings* subscale (α = 0.87; e.g., ‘When I’m feeling bad, I get confused about what emotion it is.’), the 4-item *Difficulty Identifying Positive Feelings* subscale (α = 0.89; e.g., ‘When I’m feeling good, I can’t make sense of those feelings.’), and the 8-item *Externally Oriented Thinking* subscale (α = 0.90; e.g., ‘It’s not important for me to know what I’m feeling.’). Items were rated on a 7-point Likert-type scale, with higher scores indicating more difficulties with emotion identification. The PAQ was selected instead of the more commonly used Toronto Alexithymia Scale [42] due to the PAQ’s consideration of both negative and positive feelings.

#### Difficulties in Emotion Regulation Scale– Short Form (DERS-SF)

Participants completed the Difficulties in Emotion Regulation Scale– Short Form (DERS-SF [23, 43]), including the 3-item *Lack of Emotional Clarity* subscale (α = 0.81; e.g., ‘I have no idea how I am feeling.’), the 3-item *Lack of Emotional Awareness* subscale (α = 0.55; e.g., ‘I pay attention to how I feel.’), the 3-item *Nonacceptance of Emotional Responses* subscale (α = 0.89; e.g., ‘When I’m upset, I become irritated with myself for feeling that way.’), the 3-item *Limited Access to Emotion Regulation Strategies* subscale (α = 0.77; e.g., ‘When I’m upset, I believe there is nothing I can do to make myself feel better.’), the 3-item *Difficulty Engaging in Goal-Directed Behavior* subscale (α = 0.91; e.g., ‘When I’m upset, I have difficulty focusing on other things.’), and the 3-item *Impulse Control Difficulties* subscale (α = 0.90; e.g., ‘When I’m upset, I lose control over my behavior.’). Items were rated on a 5-point scale, with higher scores indicating more difficulties with emotion perception and valuation. 

#### Emotion Regulation Questionnaire (ERQ)

The ERQ [44] was administered to measure participants’ habitual use of specific ER strategies, including the 6-item *Cognitive Reappraisal* subscale (α = 0.88; e.g., ‘I control my emotions by changing the way I think about the situation I’m in.’) and the 4-item *Expressive Suppression* subscale (α = 0.77; e.g., ‘When I am feeling negative emotions, I make sure not to express them.’). Items were rated on a 7-point Likert-type scale, with higher scores indicating more use of the target ER strategy. As all other scales in this study are scored such that higher scores indicate more ER difficulties, we reverse-coded scores on this scale to facilitate clearer interpretation of results.

#### Ruminative Responses Scale (RRS)

The 5-item *Brooding Rumination* subscale of the RRS [45] measured participants’ tendency to engage in the moody rumination at the core of Nolen-Hoeksema’s response styles theory [46] (α = 0.84; e.g., ‘When I feel down, sad, or depressed, I… think “what am I doing to deserve this?”’). Items were scored on a 4-point scale, with higher scores indicating more frequent brooding. 

### Depressive symptoms

The 20-item Center for Epidemiologic Studies– Depression Scale (CES-D [47]) measured participants’ depressive symptoms in the past week (α = 0.92; e.g., ‘I thought my life had been a failure.’). Items were rated on a 4-point scale with higher scores indicating more frequent depressive symptoms. The CES-D was administered in both Wave 2 (along with the ER measures) and Wave 3 (one year later). 

### Data analytic plan

Data were analyzed using Mplus, version 8. To examine potential differences in the prevalence of ER components by sexual orientation (heterosexual vs. sexual minority), we first ran descriptive statistics and conducted independent samples *t*-tests. We then ran two separate mediation analyses. In the first, we entered Wave 2 sexual orientation (0 = heterosexual, 1 = sexual minority) as the predictor, Wave 3 depressive symptoms as the outcome, and each of the 12 ER components (Wave 2) as independent mediators. Age and assigned sex at birth were included as covariates given their well-documented associations with ER [48–50]. We also controlled for Wave 2 depression symptoms, consistent with the longitudal mediation aims of this study. In the second, we conducted a multiple mediation analysis in which all 12 ER components were entered simultaneously into a multiple mediation model to determine which remained significant mediators when accounting for shared variance with the other ER components and controlling for age and assigned sex. All mediation models were estimated with maximum likelihood and robust standard errors. As a sensitivity analysis, we bootstrapped standard errors with 10,000 samples for the main mediation model and results were identical.

Weights were employed in the mediation analyses to make the cohort representative of Sweden’s population. Weights were derived using the process detailed by Chen et al. [51], such that weights from Wave 2 of the PLUS study were used as the base weight, and the chi-square automatic interaction detection (CHAID) algorithm was used to account for attrition at Wave 3. The resulting weights were post-stratified using the joint distribution of age and assigned sex at birth from 2020 Swedish census data.

## Results

### Sexual orientation differences in ER and depressive symptoms

As shown in Table 2, independent samples *t*-tests revealed significant differences in ER components at Wave 2 between heterosexual and sexual minority participants. Specifically, sexual minority individuals, compared to heterosexual individuals, reported significantly more difficulty with: ability to identify negative (Hedge’s *g* = 0.31) and positive emotions (Hedge’s *g* = 0.33); emotional clarity (Hedge’s *g* = 0.37); accepting their emotional responses (Hedge’s *g* = 0.40); access to emotion regulation strategies (Hedge’s *g* = 0.42); engaging in goal-directed behaviors when experiencing strong emotions (Hedge’s *g* = 0.41); impulse control difficulties (Hedge’s *g* = 0.24); using cognitive reappraisal (Hedge’s *g* = 0.14); and brooding rumination (Hedge’s *g* = 0.41). There were no significant differences between sexual orientation groups in externally oriented thinking, emotional awareness, or expressive suppression.

**Table 2:** Descriptive statistics and comparisons of emotion regulation components and depressive symptoms by sexual orientation group

	Heterosexuals		Sexual Minorities				
	*n*	Mean (SD)	*n*	Mean (SD)	t	df	Hedges’ g
**Emotion Regulation Components**							
Difficulty identifying negative feelings^1^	660	11.29 (5.87)	533	13.14 (6.32)	−5.20***	1099.91	0.31
Difficulty identifying positive feelings^1^	660	8.56 (5.13)	533	10.40 (6.12)	−5.54***	1037.22	0.33
Externally oriented thinking^1^	660	23.08 (10.60)	532	23.80 (10.62)	−1.17	1190	0.07
Lack of emotional clarity^2^	664	5.92 (2.59)	536	6.92 (2.87)	−6.26***	1089.41	0.37
Lack of emotional awareness^2^	664	8.00 (2.35)	535	8.13 (2.48)	−0.90	1197	0.05
Nonacceptance of emotional responses^2^	664	6.65 (3.27)	535	8.04 (3.75)	−6.77***	1066.52	0.40
Limited access to emotion regulation strategies^2^	664	5.74 (2.54)	535	6.87 (2.93)	−7.08***	1063.87	0.42
Difficulty engaging in goal-directed behavior^2^	664	7.91 (3.33)	535	9.29 (3.47)	−7.00***	1197	0.41
Impulse control difficulties^2^	664	4.99 (2.59)	535	5.67 (3.03)	−4.08***	1054.80	0.24
Cognitive reappraisal^3^	664	3.54 (1.20)	537	3.71 (1.24)	−2.47*	1199	0.14
Expressive suppression^3^	663	3.51 (1.31)	537	3.65 (1.33)	−1.73	1198	0.10
Brooding rumination^4^	664	10.20 (3.65)	539	11.72 (3.84)	−7.01***	1201	0.41
**Depressive symptoms** ^**5**^							
Wave 2	667	15.04 (10.33)	541	21.02 (12.20)	−9.06***	1060.11	0.53
Wave 3	667	14.69 (10.23)	541	20.15 (12.21)	−8.30***	1053.65	0.49

Note. *p <.05, **p <.01, ***p <.001. ^1^Perth Alexithymia Questionnaire [41]. ^2^Difficulties in Emotion Regulation Scale– Short Form [23, 43]. ^3^Emotion Regulation Questionnaire [44]. Scores on the cognitive reappraisal scale were reverse coded. ^4^Ruminative Responses Scale– Brooding [45]. ^5^Centers for Epidemiological Studies– Depression Scale [47]. Wave 3 depression symptoms were collected one year after all other measures. Higher scores on all measures reflect higher levels of emotion regulation difficulties or depressive symptoms. All participants included in this table completed both Wave 2 and Wave 3 of data collection

Sexual minorities also reported significantly higher levels of depressive symptoms at both Wave 2 (Hedge’s *g* = 0.53) and Wave 3 (Hedge’s *g* = 0.49) compared to heterosexuals.

### ER as a mechanism explaining sexual orientation disparities in depressive symptoms

**Fig. 1:**
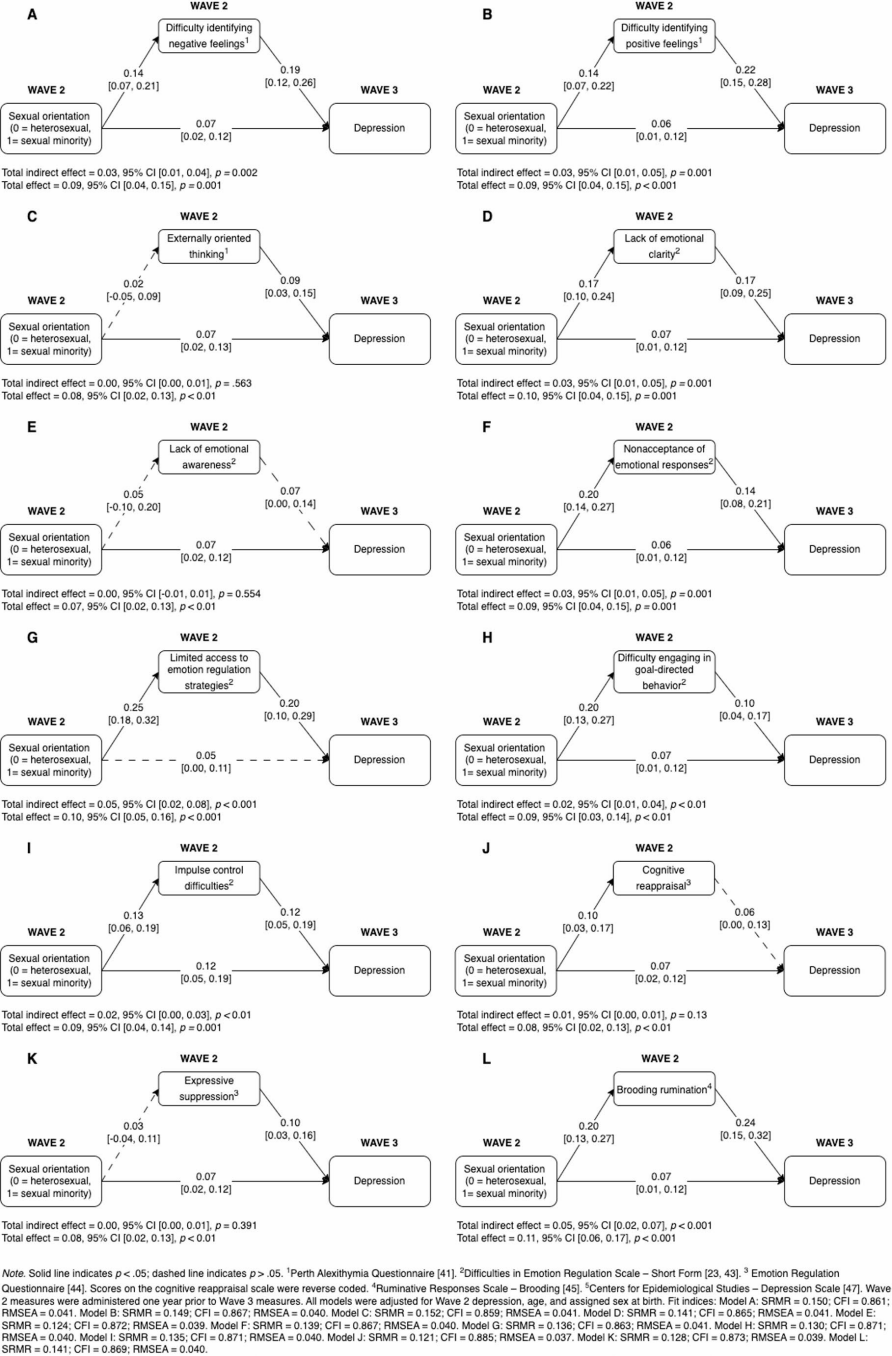
Mediation models predicting depressive symptoms from sexual orientation and each individual emotion regulation component

Table 3 presents the correlations between ER and depressive symptoms. The first mediation model examined the indirect effect of sexual orientation on depressive symptoms via each of the individual ER components (Fig. 1). Eight of the 12 ER processes independently mediated the prospective association between sexual orientation and depressive symptoms. Lack of emotional awareness, externally oriented thinking, cognitive reappraisal, and expressive suppression were not significant mediators.

**Table 3:** Pearson moment correlations between emotion regulation components and depressive symptoms

	2	3	4	5	6	7	8	9	10	11	12	13	14
**Emotion regulation components**													
1. Difficulty identifying negative feelings^1^	0.64***	0.47***	0.66***	0.23***	0.51***	0.49***	0.37***	0.42***	0.22***	0.33***	0.49***	0.46***	0.40***
2. Difficulty identifying positive feelings^1^	–	0.50***	0.57***	0.28***	0.41***	0.47***	0.33***	0.38***	0.24***	0.34***	0.41***	0.47***	0.46***
3. Externally oriented thinking^1^		–	0.46***	0.55***	0.25***	0.25***	0.09**	0.17***	0.16***	0.58***	0.19***	0.29***	0.25***
4. Lack of emotional clarity^2^			–	0.25***	0.49***	0.53***	0.38***	0.43***	0.27***	0.35***	0.54***	0.57***	0.46***
5. Lack of emotional awareness^2^				–	0.10**	0.07*	−0.06*	0.06*	0.20***	0.37***	0.07*	0.19***	0.16***
6. Nonacceptance of emotional responses^2^					–	0.62***	0.50***	0.51***	0.24***	0.27***	0.62***	0.56***	0.46***
7. Limited access to emotion regulation strategies^2^						–	0.61***	0.59***	0.36***	0.27***	0.61***	0.65***	0.52***
8. Difficulty engaging in goal-directed behavior^2^							–	0.58***	0.24***	0.10**	0.49***	0.48***	0.40***
9. Impulse control difficulties^2^								–	0.28***	0.10**	0.48***	0.50***	0.41***
10. Cognitive reappraisal^3^									–	0.08**	0.27***	0.30***	0.25***
11. Expressive suppression^3^										–	0.22***	0.29***	0.25***
12. Brooding rumination^4^											–	0.66***	0.54***
**Depressive symptoms**^5^													
13. Wave 2												–	0.67***
14. Wave 3													—

Note. *p <.05, **p <.01, ***p <.001. ^1^Perth Alexithymia Questionnaire [41]. ^2^Difficulties in Emotion Regulation Scale– Short Form [23, 43]. ^3^Emotion Regulation Questionnaire [44]. Scores on the cognitive reappraisal scale were reverse coded. ^4^Ruminative Responses Scale– Brooding [45]. ^5^Centers for Epidemiological Studies– Depression Scale [47]. Wave 3 depression symptoms were collected one year after all other measures. Higher scores on all measures reflect higher levels of emotion regulation difficulties or depressive symptoms. All participants included in this table completed both Wave 2 and Wave 3 of data collection

**Fig. 2:**
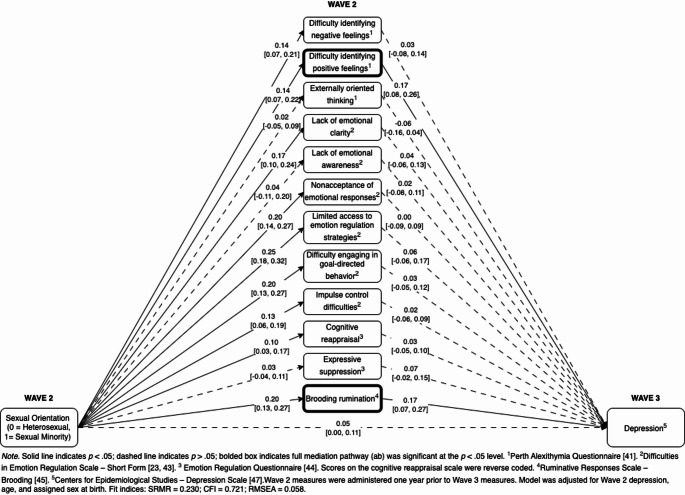
Longitudinal multiple mediation model entering emotion components as mediators between sexual orientation and depressive symptoms

In the second multiple mediation model, the 12 ER components were entered as simultaneous mediators of the prospective relationship between sexual orientation and depressive symptoms (Fig. 2). Two ER components significantly mediated this relationship: difficulty identifying positive emotions, *ab* = 0.02, 95% CI [0.01, 0.04], *p* =.006, and brooding rumination, *ab* = 0.03, 95% CI [0.01, 0.06], *p* =.003. These results indicate that sexual minorities experienced more ER difficulties in these two domains than heterosexuals, which in turn predicted an increase in depressive symptoms one year later.

## Discussion

Prior research found that stigma-related stressors predict psychopathology among sexual minorities [7] but did not elucidate the mechanisms underlying this relationship. The psychological mediation framework hypothesized, and subsequent research supported, that stigma-related experiences predict heightened ER difficulties, which in turn predict psychopathology risk [3, 11, 17, 21]. Based on that work, in this study we explored whether these ER difficulties—which result from stigma—explain (at least in part) sexual orientation disparities in mental health. Indeed, in this longitudinal, population-based sample of sexual minority and heterosexual young adults, we find evidence that sexual minority individuals have significantly more ER difficulties than their heterosexual peers across nine of the 12 ER components that we investigated, including identifying negative and positive feelings, emotional clarity, accepting their emotional responses, accessing ER strategies, engaging in goal-directed behaviors, impulse control difficulties, using cognitive reappraisal, and brooding rumination.

Our results also extend previous evidence for the psychological mediation framework [3] by demonstrating that sexual orientation differences in several ER components predict an increase in depressive symptoms one year later and underlie the well-documented disparity in depression between sexual minorities and heterosexuals [1, 2]. Specifically, we found that eight out of the 12 ER components examined herein—a substantially larger number than examined in prior work [26, 28, 29]—independently mediated the longitudinal association between sexual orientation and increases in depressive symptoms. Further, in models controlling for all ER components simultaneously, sexual orientation differences in difficulties identifying positive feelings and brooding rumination were uniquely predictive of increases in depressive symptoms. This finding aligns with prior research showing that decreased positive emotions and increased brooding rumination are associated with depressive symptoms [46, 52], as well as an emerging literature finding associations between emotion differentiation and depression [53–56]. The lack of significant mediating effects for the other ER components examined could indicate that sexual orientation differences in these ER processes are not linked to differences in depressive symptoms, even if they might be related to other mental health symptoms.

The results of this study address several important methodological gaps in the extant literature on ER and mental health in sexual minority individuals. By independently examining 12 unique ER measures, these findings expand the existing literature, which has relied primarily on non-specific composite measures of ER difficulties (e.g., DERS total score [14, 19]). While our results showed sexual orientation differences across the majority of ER components, there were some notable null results. For example, the lack of differences in emotional awareness, externally oriented thinking, and expressive suppression warrants additional research, especially given contrary results in prior studies for emotional awareness as a mediator of the sexual orientation difference in internalizing symptoms among adolescents [28]. Both externally oriented thinking and expressive suppression are forms of avoiding one’s emotions, suggesting that sexual minority individuals are no more likely to engage in experiential avoidance than their heterosexual counterparts. These findings would not have been observable if only composite measures of ER difficulties had been administered.

Additionally, our study used population-based sampling to overcome the selection biases common to the non-representative sampling methods typically used in studies of sexual minority populations [33, 35]. As a result, our findings provide important initial evidence that the sexual orientation differences in ER observed in non-representative samples [28, 57] are not merely features of those samples, but rather reflect a higher prevalence of ER difficulties among sexual minority individuals at the population level. 

### Future directions

This study’s findings offer several directions for future research. First, given the inconsistent pattern of sexual orientation differences found among ER components related to identifying emotions, whereby some were significant mediators of the sexual orientation difference in depression (e.g., difficulty identifying positive and negative feelings, emotional clarity) whereas others were not (e.g., externally oriented thinking, emotional awareness), further examination of alexithymia and its component constructs among sexual minority individuals is needed. Such investigations would benefit from experimental designs that parse alexithymia components in response to both general emotion-eliciting stimuli and those that are identity specific. In fact, theoretical accounts of sexual minority individuals’ experiences of emotions conceptualize ongoing exposure to identity-specific stress as a form of traumatic invalidation of emotional experiences and their identification [9]. Experimental tasks could also probe the specific components of emotion identification with which sexual minorities might experience particular challenges by examining both externally oriented thinking and trouble naming and identifying emotions when asked to attend to them in response to both general and identity-specific stimuli.

Second, future studies could elucidate the developmental unfolding of sexual orientation differences in ER in order to inform prevention efforts. While previous studies have documented that sexual orientation differences in certain ER processes are already apparent in young adolescents (e.g., rumination, low emotional awareness [28]), perhaps as a result of early exposure to minority stress, only a few ER processes have been examined in this age group. Prospective cohort studies using a longer time frame starting earlier in development than the present study could elucidate when in development these ER differences arise and begin to influence subsequent mental health.

Third, existing interventions, including sexual minority-affirmative cognitive-behavioral therapy [58, 59], currently emphasize emotional avoidance as one common response to minority stress [60]. However, our study did not observe sexual orientation differences in indicators of experiential avoidance, highlighting the need to develop intervention modules that target other features of ER for which sexual minorities do exhibit difficulties relative to their heterosexual peers, such as identifying positive emotions [61] and flexibly accessing a broad repertoire of ER strategies according to situational demands [62].

Fourth, given well-documented differences in the association between different types of ER and psychopathology [8], future studies should expand this investigation of the role of ER components to a broader array of mental health conditions (e.g., substance use, anxiety disorders) among sexual minorities. Through such investigations, researchers and clinicians may be able to more efficiently target particular ER components in interventions with sexual minority individuals depending on symptom presentation.

### Limitations and constraints on generality

Results of the present study should be interpreted in light of several limitations. Although the sample was representative of the young adult population in Sweden, its results may not generalize beyond Sweden’s unique cultural context. For instance, Sweden is regarded as one of the most progressive countries in the world regarding equal rights for sexual minorities, and it has been a pioneer in supportive legislation for sexual minorities (e.g., legal protection against discrimination based on sexual orientation has been in place since 1987; the right for same-sex couples to register their partnerships was established in 1995; and marriage rights for same-sex couples were granted in 2009). Swedish population attitudes towards equal rights for sexual minorities and public acceptance of homosexuality have steadily increased to now be among the highest globally [63, 64]. This improved acceptance of sexual minority individuals has been associated with a significant reduction in the magnitude of sexual orientation disparities in psychological distress in Sweden [65]. It is possible that sexual orientation differences in emotion regulation operate differently in more stigmatizing cultural contexts, a hypothesis that warrants additional study. If so, our findings may provide conservative estimates of the magnitude of sexual orientation-related ER differences.

Our results may also not fully reflect the diversity of ER experiences across the spectrum of sexual minority identities due to our decision to collapse all non-heterosexual sexual identities into a single sexual minority category in order to focus on population-level comparisons between heterosexual and sexual minority individuals. Future research should therefore examine whether the ER differences observed in this study apply across subgroups of sexual minority individuals, particularly given well-documented differences in identity-specific stress experiences by sexual orientation subgroups (e.g., bisexual individuals [66]) as well as gender differences in ER [48].

Finally, although we examined 12 ER components in this study, many others exist as described by various ER frameworks (e.g [39, 67–69]). Future work should expand our understanding of the scope of sexual orientation-related ER differences in a broader array of ER components. Such investigations should use reliable measures of ER constructs. In this study, our measure of emotional awareness had low internal reliability (also seen in other investigations with this subscale [70]), perhaps due to the challenges of self-reporting on one’s own awareness. Expanding future studies to include non-self-report measures of ER components (e.g., experimental paradigms) would further elucidate our understanding of sexual orientation-related ER differences. Relatedly, our study examined trait-level ER components, rather than ER responses to minority stress for sexual minorities, due to the methodological challenges of asking different questions of different groups (i.e., asking sexual minority participants about ER related to minority stress, questions which are not relevant to heterosexual participants). However, this decision makes it difficult to disentangle whether the observed sexual orientation differences in ER are due to stable individual difference factors or to contextual factors like minority stress that contribute to psychopathology. There is some evidence from recent research on rumination in response to identity stressors that it is the process (i.e., rumination) rather than the content (i.e., identity- vs. non-identity-related thoughts) that is most important for predicting mental health outcomes [71]. Future work is needed to examine whether the same is true for all 12 ER components examined in this study.

## Conclusion

Using a longitudinal, population-based design, the present study demonstrated that sexual minority young adults disproportionately experience a wide range of ER difficulties, many of which predicted their elevated rates of depressive symptoms as compared to their heterosexual peers. The present study joins previous research indicating that elevations in ER difficulties result from sexual minority individuals’ disproportionate exposure to stigma-related stressors [3] to call for future mechanistically informed interventions capable of improving ER in this population [3, 58, 72].

## Data Availability

All data were produced under the Swedish Statistics Act and the European Union Data Protection Regulation, according to which privacy concerns restrict the availability of personal data for research. Aggregated data can be made available by the authors, subject to ethical vetting. Enquiries should be made to plus@cns.ki.se. The code for these analyses is publicly available and can be accessed at: https://osf.io/mhsyp/?view_only=378179dda995439e975b18d1db013a20.
